# Amygdala volume and hypothalamic-pituitary-adrenal axis reactivity to social stress

**DOI:** 10.1016/j.psyneuen.2017.07.487

**Published:** 2017-11

**Authors:** Tom J. Barry, Lynne Murray, Pasco Fearon, Christina Moutsiana, Tom Johnstone, Sarah L. Halligan

**Affiliations:** a Department of Psychology, The University of Hong Kong, Pokfulam Road, Hong Kong; b Institute of Psychiatry, Psychology & Neuroscience, King’s College London, London SE5 8AF, UK; c School of Psychology and CLS, University of Reading, Reading RG6 6AL, UK; d Department of Psychology, Stellenbosch University, Stellenbosch, South Africa; e Department of Psychiatry and Mental Health, University of Cape Town, Cape Town, South Africa; f Research Department of Clinical, Educational and Health Psychology, University College London, 1-19 Torrington Place, London WC1E 7HB, UK; g Department of Psychology, University of Westminster, 115 New Cavendish Street, London W1W 6UW, UK; h Department of Psychology, University of Bath, Bath BA2 7AY, UK

**Keywords:** Depression, Stress sensitivity, Cortisol, Amygdala, Hypothalamic-pituitary-adrenal axis

## Abstract

The amygdala plays a central role in emotional processing and has an activating influence on the hypothalamic-pituitary-adrenal (HPA) axis. Structural changes in the amygdala have been associated with early adversity and, in principle, may contribute to the later emergence of emotional pathologies by influencing the way that the brain responds to stress provocation. The present study examined the relationship between amygdala volumes and cortisol secretion in response to a social stressor among young adults who were or were not exposed to maternal postnatal depression (PND) early in development (referred to as PND offspring and controls, respectively). Hierarchical Linear Modelling (HLM) revealed that, on a sample-wide level, there was no evidence of a relationship between total amygdala volume, or the volume of the right or left hemisphere amygdala taken separately, and cortisol reactivity. Unexpectedly, for PND offspring, larger right hemisphere amygdala volume was associated with lower cortisol reactivity in response to stress, an effect that was not apparent in control offspring. We conclude that the relationship between amygdala volumes and stress reactivity may not be as clear as previous models suggested.

## Highlights

• Amygdala volumes were not associated with stress-related cortisol reactivity.
• However, postnatally depressed mothers’ offspring showed an association.
• For these offspring, smaller volumes corresponded with heightened reactivity.
• This association was relative to age-matched control offspring.

## 1 Introduction

Structural alterations in the amygdala have been associated with environmental adversities during early development. Enlarged amygdala volumes have been observed in samples of orphanage reared children, where environments are characterised by neglect (Tottenham et al., 2010); and in the context of chronic maternal depressive symptoms, an association hypothesised to be due to the withdrawn parenting typical of maternal depression (Lupien et al., 2011). In our own research we found that infant attachment insecurity, which may arise as a consequence of depression-related parenting difficulties, predicted greater total amygdala volume (Moutsiana et al., 2015), but we did not find direct effects of maternal depression.

The amygdala plays a central role in emotional processing and responding, and, correspondingly, studies have linked greater amygdala volumes with negative affectivity (Holmes et al., 2012), sensitivity to negative experiences (Barros-Loscertales et al., 2006, Gerritsen et al., 2012), and elevated anxiety (Baur et al., 2012, Tottenham et al., 2010). Such associations may partly arise due to limbic system influences on the HPA axis. The amygdala can have an activating influence on the HPA stress response system (Pruessner et al., 2010, Herman et al., 2005), particularly in relation to psychological stressors (Hand et al., 2002). This effect is predominantly mediated by central or medial amygdaloid nuclei, and is part of a larger system of limbic control (Herman et al., 2005).

Despite the evidence for amygdala modulation of HPA responding, there has been limited direct examination of whether volumetric alterations in amygdala, as identified in some at risk groups of humans, are related to HPA activity, and the available evidence is mixed. A study of 24 unipolar depressed inpatients and 14 healthy controls found no association between amygdala volume and basal cortisol secretion (Kronenberg et al., 2009). A second study of 76 unipolar depressed inpatients similarly found no overall evidence that amygdala volume was related to cortisol secretion in response to the combined dexamethasone-corticotrophic releasing hormone test (although there was tentative evidence that greater amygdala volume at baseline was positively associated with normalisation of HPA response to challenge following antidepressant treatment) (Schuhmacher et al., 2012). Further, research which identified larger amygdalae in trauma exposed versus non-exposed individuals also found no association between amygdala volumes and cortisol secretion in response to dexamethasone test (Cacciaglia et al., 2017). Importantly, none of these studies examined HPA activation in response to *psychological* stress, yet activation by the amygdala may be particularly relevant in this context. One study that did investigate amygdala volumes in relation to cortisol output during the Trier Social Stress Test (TSST) found the relationship to be complex: while there was a positive correlation between amygdala volume and cortisol reactivity for adolescents with major depressive disorder (MDD), control participants showed an inverse association (Klimes-Dougan et al., 2014).

Here, we sought to examine cortisol output in response to the TSST in relation to amygdala volumes in a longitudinally studied sample of 58 young adults (Moutsiana et al., 2015), where participants were originally recruited on the basis of the presence or absence of maternal PND. Previous analyses with this sample have found maternal PND to predict elevated offspring basal morning cortisol secretion at 13-years (Halligan et al., 2004), and greater TSST cortisol reactivity at 22-years (Barry et al., 2015). We therefore took account of PND group status and other key factors (gender, overall brain volume, history of depression) in our analyses. We hypothesised that greater amygdala volumes would be associated with heightened reactivity to the TSST. Given the moderation by MDD status reported by Klimes-Dougan et al. (2014) we also tested whether maternal PND related risk of depression similarly moderated effects.

## 2 Method

The study was approved by the ethics committees of the University of Reading (08/64) and the National Health Service (08H0606115). Participants provided informed consent.

### 2.1 Participants

Participants were part of a longitudinal study examining the development of 100 children of postnatally depressed and well mothers from infancy. At age 22 years, 38 offspring of PND mothers and 38 controls were available to attend the university for a day of testing, including a structural MRI scan at 11:00 h and the TSST at 15:00 h (for details see Barry et al. (2015)). Of these, 14 participants met exclusion criteria due to medical conditions which made scanning unsafe (diabetes, epilepsy or metal implants). Loss of scanning data also occurred due to significant structural abnormalities (n = 2), poor image resolution (n = 1) and incomplete scanning (n = 1), resulting in a final sample of 58 participants, 27 PND offspring and 31 controls (age *M* = 22.4 yrs, *SD* = 0.6; females n = 28). As previously reported, there was evidence of selective attrition, with fewer PND (50.9%) versus control group (75.6%) offspring participating (χ^2^ = 5.95, *p* = 0.015) (Barry et al., 2015).

### 2.2 TSST

The TSST is a social stress test that reliably stimulates cortisol secretion. It was administered according to standard protocols, combining a 5-min mental arithmetic task and a personally relevant speech delivered to a panel of two unresponsive observers (Kudielka et al., 2007). Saliva smples were collected via passive drooling prior to TSST instructions, immediately post-test and then 10-, 20-, 30- and 45-min post-test. Samples were storeat −20C and later thawed and centrifuged at 3000 rpm for 5 min to produce clear supernatant fractions of low viscosity. Free cortisol was assayed by luminescence immunoassay (Immuno-Biological Laboratories, Hamburg, Germany). Inter- and intra-assay coefficients of variation were <7%.

### 2.3 MRI data acquisition and processing

High-resolution three-dimensional (3D) T1-weighted images were acquired on a 3-T whole-body scanner (Siemens MAGNETOM Trio) with a 12-channel Head Matrix coil. The MRI parameters of the 3D magnetization-prepared rapid gradient-echo sequence were the following: FOV = 250 × 250 mm^2^, TR/TE/TI/FA = 2020 ms/2.52 ms/1.1/9°. Images were acquired with an in-plane spatial resolution of 0.9765 mm and 176 contiguous sagittal 1 mm thick slices, generating nearly isotropic three-dimensional MR data sets for accurate volumetric MR measurements. Data processing was conducted using FMRIB’s Software Library (FSL) version 4.1.8 (www.fmrib.ox.ac.uk/fsl). Non-brain tissue was removed from the high resolution anatomical images using BET (Smith, 2002), and remaining voxels summed to give an estimate of the total intracranial volume per participant, which we used as a covariate in analyses. Volume of interest analysis was carried out using individual amygdala masks using the automated FSL tool, FMRIB’s Integrated Registration and Segmentation Tool version 1.2. Manual checks identified no segmentation errors. Non-zero voxels/volumes within the masks were calculated in mm^3^ using Fslstats (http://fsl.fmrib.ox.ac.uk/fsl/fslwiki/Fslutils).

### 2.4 Statistical analysis

Missing cortisol data (6% of samples due to insufficient saliva) were estimated using the SPSS Expectation-Maximization algorithm based on available cortisol samples. Four outliers (±3 *SD* from the mean) were excluded from analyses. Cortisol values were log transformed to reduce skew. The relationships between total amygdala volume, as well as right and left hemisphere amygdala volumes, and cortisol reactivity over the TSST were analysed using separate two-level HLMs conducted in MLwiN 2.28 (http://www.bristol.ac.uk/cmm/software/mlwin/). A level-1 model estimated the intercept and linear and quadratic slopes of change of log transformed cortisol reactivity over the TSST, with times coded as decimals of the actual time in minutes. Level-2 models estimated the variance in the intercept and slopes that were predicted by person-level differences in total and right and left hemisphere amygdala volume separately. The effects of gender and whole brain volume were controlled for due to established relationships with cortisol reactivity and amygdala volume. We also examined whether maternal PND moderated any of the effects of amygdala volume on cortisol reactivity, by including PND group status (present/absent) in level 2 models as a main effect and in interaction with amygdala volumes.

## 3 Results

Sample characteristics are presented in Table 1. The level-1 model evidenced sufficient variation in cortisol parameters between participants to support modelling as a function of other variables (see Barry et al., 2015).

**Table 1:** Participant characteristics.

	Whole Sample N = 58	PND n = 27	Control n = 31
Age, *M(SD)*	22.4 (.6)	22.4 (0.6)	22.4 (0.7)
Proportion of males	51.7%	48.1%	54.8%
Proportion with a history of depressive disorder^a^	29.3%	40.7%	19.4%+
Proportion with current medication usage^b^	12.1%	11.1%	12.9%
Depressive symptoms (CESD), *M(SD)*	10.6 (9.8)	10.6 (9.2)	10.7 (10.5)

Raw cortisol volumes (nmol/l)			
Pre-test, *M(SD)*	7.0 (3.1)	6.5 (2.1)	7.5 (3.7)
Post-test +0, *M(SD)*	9.0 (4.4)	9.5 (4.5)	8.5 (4.3)
Post-test +10, *M(SD)*	9.2 (4.4)	9.9 (4.6)	8.6 (4.3)
Post-test +20, *M(SD)*	11.7 (6.2)	12.0 (5.3)	11.4 (7.0)
Post-test +30, *M(SD)*	10.6 (5.2)	10.6 (4.3)	10.6 (5.9)
Post-test +45, *M(SD)*	8.9 (4.7)	9.0 (4.6)	8.8 (4.8)

CESD: Centre for Epidemiological Studies Depression Scale.

+ *p <* 0.10.

a Measured using the Structured Clinical Interview for DSM-IV at 22 years, and the Kiddie Schedule for Affective Disorders and Schizophrenia at ages 8, 13, and 16 years (see Barry et al. (2015) for details; participants were not tested during a current episode of major depression at 22-years).

b Only medication known to influence cortisol activity is included: one participant from each group was taking antidepressants; two participants from each group were taking a contraceptive pill; and one control participant was taking thyroid medication. Three participants reported smoking.

### 3.1 Cortisol reactivity and amygdala volumes

Cortisol reactivity over time was modelled as a function of amygdala volume, controlling for the effects of gender and whole brain volume (see Table 2). Neither total amygdala volume, nor right or left hemisphere amygdala volumes predicted significant variance in the intercept or the linear or quadratic slopes for cortisol reactivity over time. As previously reported for these data (Barry et al., 2015), gender predicted a significant amount of the variance in the linear and quadratic slopes of cortisol reactivity over time, such that males showed greater cortisol reactivity than females.

**Table 2:** Results of hierarchical linear modelling of cortisol responses to the Trier Social Stress Test.

Model	Model effects					
	Intercept		Linear		Quadratic	
	B(SE)	χ^2^(df = 1)	B(SE)	χ^2^(df = 1)	B(SE)	χ^2^(df = 1)
Total amygdala volume	0.26 (1.47)	1.11	−0.38 (0.78)	0.24	0.02 (0.14)	0.02
Gender^2^	−1.25 (1.28)	0.95	−1.93(0.68)	8.15**	0.33 (0.12)	7.28**
Total brain volume	−5.15 (5.38)	1.79	4.16 (2.83)	2.15	−0.46 (0.51)	0.84

Right hemisphere amygdala volume	1.54 (1.98)	0.44	−0.70 (1.05)	0.51	0.09 (0.19)	0.63
Gender^2^	−1.18 (1.28)	0.85	−1.93 (0.67)	8.23**	0.33 (0.14)	7.53**
Total brain volume	−5.24 (5.33)	0.97	4.08 (2.82)	2.10	−0.47 (0.50)	0.87

Left hemisphere amygdala volume	−2.44 (3.02)	0.66	0.01 (1.59)	0.00	−0.13 (0.28)	0.20
Gender^2^	−1.41 (1.28)	1.22	−1.88 (0.67)	7.81**	0.32 (0.12)	6.95**
Total brain volume	−4.24 (5.41)	0.61	3.98 (2.86)	1.94	−0.41 (0.51)	0.66

** *P *< 0.01; ^2^0 = male, 1 = female.

### 3.2 Interaction and sub-group analyses

Building on the above models, we examined PND group status as a potential moderator of outcomes. There was no significant interaction between PND group status and total amygdala volume for the cortisol intercept, *B* = −3.69, *SE* = 2.78, χ^2^(1) = 1.76, *p* = 0.18. However, there were significant PND by amygdala interactions in relation to both linear, *B* = −3.11, *SE* = 1.49, χ^2^(1) = 4.34, *p* = 0.037, and quadratic slopes, *B* = 0.57, *SE* = 0.27, χ^2^(1) = 4.65, *p* = 0.031. Post hoc, within group analyses indicated that for the PND group, there were non-significant trends for amygdala volume to predict the intercept, *B* = −2.85, *SE* = 1.58, χ^2^(1) = 3.24, *p* = 0.072, the linear slope, *B* = −1.83, *SE* = 0.98, χ^2^(1) = 2.78, *p* = 0.061, and the quadratic slope, *B* = 0.31, *SE* = 0.18, χ^2^(1) = 3.08, *p* = 0.079. In the control group there were no significant associations between amygdala volume and any cortisol estimate (all *p >* 0.186).^1^

Follow up analyses examined amygdala volumes by laterality. For right amygdala volume, the PND by amygdala volume interaction term was a significant predictor of both the linear slope, *B* = −5.20, *SE* = 2.06, χ^2^(1) = 5.76, *p* = 0.012, and the quadratic slope, *B* = 0.93, *SE* = 0.37, χ^2^(1) = 6.46, *p* = 0.011, but not the cortisol intercept, *B* = −3.51, *SE* = 3.87, χ^2^(1) = 0.82, *p* = 0.37. Again, post-hoc within group analyses indicated that for the PND group, right amygdala volume was not a predictor of the cortisol intercept, *B* = −1.37, *SE* = 2.36, χ^2^(1) = 0.34, *p* = 0.56, but was significantly associated with the linear slope, *B* = −3.51, *SE* = 1.35, χ^2^(1) = 6.76, *p* = 0.009, and the quadratic slope, *B* = 0.62, *SE* = 0.24, χ^2^(1) = 6.56, *p* = 0.010. By contrast, in the control group there were no significant associations between right amygdala volume and any cortisol estimate, all *p >* 0.22. Analyses of left amygdala found no evidence of an interaction between volume and PND exposure in relation to any component of cortisol output, all *p >* 0.21.

## 4 Discussion

Several existing studies, including our own, have indicated enlarged amygdalae in association with less optimal early environments. However, there has been limited investigation of the potential functional significance of such findings. We sought to address this gap by examining whether there was a positive association between amygdala volume and HPA reactivity in response to a social stressor. Contrary to expectations, on the whole-sample level there was no evidence of a relationship between total amygdala volume, or the volume of the right or left hemisphere amygdala taken separately, and cortisol reactivity to the stressor. Secondary analyses provided tentative evidence of an effect of PND exposure on the relationship between amygdala volume and cortisol reactivity. However, the effect identified was not in the predicted direction – for PND offspring (but not controls), volume of the right hemisphere amygdala was *negatively* associated with cortisol reactivity.

Our overall null findings regarding amygdala volume and cortisol reactivity confirm and extend previous findings that amygdala volumes were not related to basal levels of cortisol (Kronenberg et al., 2009) or HPA activity in response to a biological challenge (Schuhmacher et al., 2012, Cacciaglia et al., 2017). However, subgroup analyses yielded effects that were less consistent with existing findings. For control group offspring, there was no relationship between amygdala volumes and reactivity, but for PND offspring, larger right amygdala volumes were associated with reduced linear and increased quadratic slopes for cortisol reactivity over time, that is, reduced increase and faster recovery. This contrasts to previous observations that larger amygdala volumes predicted increased TSST cortisol output for depressed adolescents, but reduced output in non-depressed controls (Klimes-Dougan et al., 2014). It is possible that analytic differences contributed to the discrepant findings. Thus, we used HLM to model parameters for cortisol starting value, reactivity and recovery, whereas Klimes-Dougan et al. (2014) focused on area under the curve relative to ground, which captures total hormonal output well, but is highly correlated with basal secretion and may index reactivity less precisely. Notwithstanding these differences, it remains difficult to account for the reversed direction of effects across studies. The effect that we observed in the PND group was not accounted for by their own history of depression, but could, in principle, be a function of developmental exposure to maternal disorder (in utero, postnatally or subsequently) or underlying genetic factors.

Although comparable in size to previous studies, the current study had limited power to detect effects. Moreover, we found evidence of selective attrition (Barry et al., 2015), highlighting the potential for bias in our study and in the wider psychobiological literature, where recruitment percentages are not routinely considered. Nonetheless, our findings and those of others suggest that the link between amygdala volumes and HPA reactivity may not be as clear as the equivalent, positive association found for amygdala activity (Weldon et al., 2015, Klimes-Dougan et al., 2014, Taylor et al., 2008). Of course, MRI based measures of neuroanatomy do not directly measure the cellular compartments that determine neural signalling (Lerch et al., 2017). Moreover, the amygdala may particularly be involved in responding to fear stimuli rather than the psychological stress imposed by the social provocation used here (Pruessner et al., 2010, Herman et al., 2005). Examination of a wider range of amygdala structural properties and stress responses could be informative.

Our overall null findings regarding the sample-wide association between amygdala volumes and HPA reactivity, and the unexpected pattern of interactions regarding PND exposure, highlight the critical need for volumetric amygdala alterations to be investigated further before conclusions are drawn regarding their significance. It is notable that although several studies have demonstrated enlarged amygdalae in association with less optimal early environments, there is also evidence that *smaller* amygdalae may be present in stress-related psychopathologies such as posttraumatic stress disorder (Morey et al., 2017). More broadly, in the absence of benchmarks for what is pathological in terms of amygdala volumes (or HPA stress response), it is essential to establish the functional significance of findings, as some effects identified in at-risk or clinical groups may reflect adaptation versus dysfunction.

## Footnotes

1 As there was a trend (p = 0.074) for lifetime depressive disorder to be more prevalent in PND versus control participants, we investigated whether this potentially accounted for effects. First, we substituted participant depression history present/absent for PND status in analyses of total amygdala volume and found no evidence that offspring depression status moderated any association between amygdala volume and cortisol output. Second, we included offspring depression status as a covariate in PND focused moderator analyses and confirmed that findings were unchanged. No participant was in a current episode of major depression at the time of testing.
